# Using partnership approach to reduce mortality and morbidity among children under five in Limpopo province, South Africa

**Published:** 2012-12-26

**Authors:** Kennedy Sivhaga, Boniface Hlabano, Penina Ochola Odhiambo

**Affiliations:** 1 Africa Medical and Research Foundation (AMREF), South Africa

**Keywords:** Integrated community management of childhood illnesses, community health workers, partnerships

## Abstract

**Background:**

Limpopo province has among the highest child mortality and morbidity rates in South Africa. To address this problem, the African Medical and Research Foundation implemented an integrated c-IMCI and child survival project. This paper reports the organization's experience in the project.

**Methods:**

AMREF South Africa implemented and tested a replicable approach for integrating health systems c-IMCI in a child survival project. The project was implemented in Limpopo province, Makhuduthamaga local municipality which is one of the most disadvantaged and under-resourced areas in South Africa. The project was implemented through a partnership model that included government departments, NGOs, CBOs, local government, traditional leaders, traditional healer's practitioners, mothers of children under five and other community structures. Monitoring and evaluation data was collected monthly and quarterly from the households of mothers of children under five by trained community health workers and their coordinators. Data regarding the performance on key child health indicators was obtained from health facilities.

**Results:**

There was improved performance in health indicators for diarrhoea incidence which dropped to 5/1000 from the baseline figure of 54/1000. Immunisation coverage improved by 11%. Vitamin A coverage for children under 1 year rose to 45% from a baseline of 27.2%. The proportion of facilities providing integrated management of childhood illness in the district rose to 100% from an initial 75%. Rate of HIV positivity among under-fives dropped to 12% from 17%.

**Conclusion:**

Use of partnerships through structures such as project task teams and Project steering committee is fundamental in ensuring good progress in reduction of diarrheal incidence, immunization coverage, sustainability and ownership of interventions.

## Background

Limpopo is one of the most disadvantaged and under-resourced provinces in South Africa. About 80% of the households in Limpopo province live below the poverty line, the highest rate in South Africa [1]. Children in Limpopo often die at home with little or no contact with the formal health service. These deaths are largely as a result of HIV and AIDS and preventable diseases like diarrhoea, pneumonia, malnutrition, malaria and other respiratory infections. According to the Joint United Nations Programme on HIV and AIDS (UNAIDS) child survival prospects remain dismal in Africa given the HIV and AIDS pandemic [2]. It is indicated that Sub-Saharan children who die before the age of five years are often characterized by poverty, illiteracy, the burden of occupational and communicable diseases, large family size, poor immunization coverage rates and poor access to basic health and socio-economic services. In Sub-Saharan Africa under five mortality is two to three times greater compared to any other region in the world. Under five mortality is over 200 per 1000 live births in the Democratic Republic of Congo, Burkina Faso, Mali, and the Central African Republic, and it reaches 259 deaths per 1000 live births in Niger [3].

It is estimated that 60% of deaths of under fives can be prevented through the use of Community Based Interventions, Health promotions and preventions by Community Health Workers (CHWs) working at the households level [4]. The IMCI approach has been piloted as a way of addressing childhood illnesses in South Africa but c-IMCI has not been adequately scaled up. At the start of the project, Community Health Workers were not sufficiently linked to the health systems. Little research has been conducted to investigate the value of using the partnership approach in reducing child mortality and morbidity in rural areas.

According to the State of the World's children report published by UNICEF in 2008, partnership holds great promise for accelerating progress towards the achievement of MDGs [5]. The study indicates that UNICEF is also working closely with UN partners, government, regional and non-governmental foundations and private sectors to coordinate activities and to pool expertise and knowledge. The report noted that UNICEF's challenge now is “to act with a collective sense of urgency to scale up that which has proven successful”. Emphasis on partnership alliances has become more and more critical in recent times because it enables each partner to bring their comparative advantage on the table to improve program quality and reach.

AMREF with a grant from UBS-Optimus Foundation initiated Makhuduthamaga Child Survival Project (MCSP) in 2008 - 2011. The overall objective of the project was to reduce mortality and morbidity among children under five in Sekhukhune district, Limpopo province, South Africa. With this project, AMREF became the second organisation to implement c-IMCI in a rural setting of South Africa.

## Methods

### The LIDS model

The model of linking communities with the District health systems (LIDS) was applied. LIDS is a model that AMREF has used over the past in Countries where it works in Africa, and has built the capacity of midlevel managers in other countries of Africa on a consultancy basis. By using LIDS in the countries of operation and training others in its use, AMREF has reported positive health outcomes and similar experiences were observed by Baqui et al (2008) [6]. This is because community based health care systems (services and outreach) and participation is the basis of addressing health barriers. The approach links families to health services in a participatory and user friendly way. In this project, the model was used to strengthen the partnership alliances to increase community participation and ownership.

### Composition of the collaborative partners

Partners for the Makhuduthamaga Child Survival Project included Department of Health; Department of Social and development (SOCdev) - District SOC-dev and sub-district SOC-dev for Makhuduthamaga and Child Care Forums (CCFs); South African police services (SAPS); South African Social and Security Agency (SASSA); Home Affairs; and Makhuduthamaga local municipality - community service unit. NGOs and CBOs were comprised of MK Umbrella, Sekhukhune Edu-care project (SEP) and other 33 Community Based Organisations (CBOs) mentored by MK Umbrella. Traditional Healers Forums, Traditional leaders, Faith Based Organisations (FBOs), politicians were collaborative partners implementing Makhuduthamaga Child Survival Project. All these partners were assembled together and their managers formed a Task Team. A Project Steering Committee (PSC) was also established at operational level and it comprised of a nurse (focal person), CHWs, Traditional Healers, Faith Based Organisations and traditional leaders. A monitoring and evaluation sub-committee was also formed. It comprised of AMREF Project manager, AMREF Monitoring and Evaluation Manager, MK Umbrella projects coordinator, DOH local area manager from Makhuduthamaga sub-district and deputy manager from Sekhukhune MCWH&N.

### Major functions of structures established

The task team was established in order to provide strategic guidance and monitoring of project performance. The project steering committees role was to monitor day to day implementation of activities. The monitoring and evaluation sub-committee developed and reviewed project monitoring tools and reported back to the task team, whereas Child Care Forums (CCFs) identified orphans and vulnerable children and referred them to relevant government departments according to their needs.

### Monitoring and evaluation

Quantitative methodologies were employed during the implementation of the project. Monitoring and evaluation tools were developed in partnership with the Department of Health and MK Umbrella to collect data from the households of mothers of under fives. Data was collected on monthly and quarterly basis by 156 trained Community Health Workers (CHWs) and their coordinators using a monthly tool and household card piloted by Provincial Directorate on Maternal, Child, Women, Health and Nutrition (MCWH&N). Data collected by trained CHWs was submitted to the Projects coordinator working for MK Umbrella who normally recorded it in the database and then produced reports for partners.

### Training on c-IMCI

In this Makhuduthamaga Child Survival Project, members of the PSCs were trained for 3 days on c-IMCI and the content of the training included identification of the sick child, knowledge of 17 key family practices and the referral systems.

### Visitation to households

Community health workers visit the households everyday and educate mothers of under five, caregivers or significant others who remained with the child about the importance of understanding 17 key family practices. The households visit by CHWs is more crucial in the sense that they are able to report back to their CBOs who then report to MK Umbrella (AMREF Partner) and to the department of Health. The reports are screened and discussed at the PSC level and Task team level. The Task team then provided strategic guidance to the implementer about the direction of the project.

## Results

Data from 18 clinics indicated improved performance with regard to priority indicators for monitoring Makhuduthamaga Child Survival Project (Table 1). There was demonstrable reduction of more than 5/1000 in the incidence of diarrhoea. Vitamin A for 1-4 years indicates the target of 40% and progress demonstrates 45% increase coverage. Pneumonia incidences indicate 5/1000 target and performance shows 23/1000. With Immunization coverage for under 1 year, the target set was 90% coverage and the results showed 72% progress which is below the target. The main challenge raised for this result was the inadequate distribution of new vaccines. The weighing rate for children was targeted at 90%. However, results showed a rate of 72%. The main challenge raised in this respect was poor recording of childrens weight by nurses attending to children at the clinic. The project also targeted 100% increase in the number of facilities implementing c-IMCI, and performance showed an increase from 0% to 100%. Within the period of 3 years of implementation, 14,400 children under five were targeted by the project; however, trained CHWs from 28 CBOs managed to reach 20,194 children.

**Table 1:** Achievements on priority indicators after three years of project implementation

Indicator	Baseline	Required target	Achievement
**Pneumonia incidence of <5 yrs**	59/1000	5/1000	23/1000
**Diarrhoea incidence under 5 yrs**	54/1000	5/1000	5/1000
**Immunization coverage <1 yr**	61%	90%	72%
**Weighing rate**	82%	90%	71.40%
**Vitamin A coverage**	27.20%	40%	45%
**% of facilities fully implementing IMCI strategy**	75%	100%	100%
**HIV+ rate <5**	17	10	12 (raw data)
**% of facilities implementing household & c-IMCI**	0%	100%	100%

## Discussion

Using partnership approach in reducing child mortality and morbidity amongst under five children strengthens the referral system amongst key role players who are working for a common goal. This was mainly a result of the referral responsibilities played by the trained PSC members.

In resource-limited settings, particularly were Community Health Workers are not consistently paid stipend, it becomes a challenge for them to volunteer their services consistently. However, in Makhuduthamaga Child Survival Project, AMREF trained a group of Community Health Workers, PSC members, Child Care Forums on c-IMCI and they received certificates of attendance as a way of motivating them and recognising their key role in addressing child health problems. This in a way contributed to their continued participation in the project activities.

The role played by trained CHWs in visiting households of children under five is very critical in addressing child mortality and morbidity. Allocating the trained CHWs households of under five children from their respective villages of their residents increases the CHWs efficiency.

The household visits by CHWs enable collection of vital information that is needed in monitoring of child survival. The reporting chain that works best is for the CHWs to report back to their CBOs who in turn report to MK Umbrella (AMREF Partner) and finally to the department of Health.

Child Care Forums (CCFs) under the chair of a Social worker ensures that under five children receive government services such as child support grant and foster care grant to those who qualify. The chair is also in a very good position to monitor whether such money is timely received and used for the intended purpose.

During the implementation of the Makhuduthamaga Child Survival Project, mothers of children under five were included in structures such as CCFs, Task teams and PSC. This was a very effective strategy as they play a vital role in mobilising other mothers during door to door campaigns and in educating others about the importance of 17 key family practices. This approach also promotes project ownership by the community. Consequently, sustainability is enhanced.

Training of the traditional healers in c-IMCI and their inclusion in the PSC and the Task team contributed in increased referrals cases and good working relations with health facility workers.

## Conclusion

Child mortality and morbidity amongst under five children is caused by preventable and treatable diseases worldwide, however, momentum to control it using partnership approach seemed to be effective. The partnership approach should become a cornerstone solution to inspire action by health policy makers especially in Africa. Now is the time for advocates, scientists, academics, practitioners, health and development officials and donors to join their voices. Together, we can build momentum and overcome the devastating toll that preventable and treatable diseases take on children, families, and communities around the world and Africa in particular. More evidence on the effectiveness of using partnerships to improve health outcomes in communities through for example cluster randomised trials is recommended.
